# Complexation of phycocyanin with hydroxypropyl-β-cyclodextrin and its application in blue beer containing quinoa saponins as foaming agents

**DOI:** 10.3389/fnut.2023.1209193

**Published:** 2023-07-13

**Authors:** Guangjie Zhang, Hongmei Zhao, Tianzhu Guan, Zheng Ma

**Affiliations:** ^1^School of Biology and Food Engineering, Anyang Institute of Technology, Anyang, China; ^2^School of Food Science and Engineering, Yangzhou University, Yangzhou, China; ^3^Department of Thoracic Surgery, Qilu Hospital of Shandong University, Jinan, China

**Keywords:** inclusion complex, phycocyanin, hydroxypropyl-β-cyclodextrin, processing stability, blue beer

## Abstract

**Introduction:**

With the increasing importance attached to human health, the inclusion complex (IC) of phycocyanin (PC) into hydroxypropyl-β-cyclodextrin (HP-β-CD) have been devoted to developing the use of food preservation in this study.

**Methods:**

In this experiment, the IC of PC into HP-β-CD was prepared by the freeze-drying method and characterized by OM, TEM, UV, FTIR and TG/DSC methods.

**Results and discussion:**

The spectroscopic features were evaluated by Ultraviolet-visible (UV-vis) spectroscopy and Fourier transform infrared spectroscopy (FT-IR) confirming that PC was located in the hydrophobic cavity of HP-β-CD. Consistent with the structural properties, optical microscopy (OM) and Transmission electron microscope (TEM) observed that the addition of PC subjected the IC to an aggregation state with irregular lamellar structures. Stability assessment showed that pH, heat and light tolerance of PC significantly regulated and improved due to the PC/HP-β-CD complexation. The formation of ICs was helpful to enhancing the antioxidant activity of PC. Molecular modeling suggested that the D-pyrrole ring and its associated C=C group of phycocyanin entered the HP-β-CD cavity from the wider edge. On this basis, the development of blue beer with quinoa saponins as foaming agent and ICs as colorant was explored. The addition of quinoa saponins made the foam richer and more delicate without destroying the overall taste coordination of beer. Moreover, the protective effect of HP-β-CD presents a positive impact on the stability of blue beer pigment. Hence, PC encapsulated into HP-β-CD will be an impressive approach in food-related application of PC.

## 1. Introduction

As a water-soluble, natural, and non-toxic molecule, phycocyanin has been obtained from the spirulina blue-green algae with intense blue color and fluorescent properties (1). Phycocyanin (PC), having chemical formula C_33_H_38_N_4_O_6_ and molecular weight of approximately 112 kDa, consists of α- and β-chain type globin, which covalently bound to phycobilin by thioether linkage (2). The PC complex presents different λ_max_ from pH 5 to pH 7 and pH sensitivity (3). Reported health benefits of PC consumption include cardioprotective, anti-inflammatory, cholesterol-lowering, anti-tumor, and neuroprotective effects (4). As a marine natural pigment, PC is extensively applied for supplementation and food colorant. Spirulina powder containing PC was blended into the brewing process of beer to develop blue beer (5). However, the major problems of this phytochemical are its low thermal stability and light and thermal instability, which severely restrict its applications. Encapsulating in cyclodextrins (CDs) could overcome these disadvantages.

Cyclodextrins (CDs) are one of the simplest encapsulant systems in the pharmaceutical and food industries. CDs are cyclic oligosaccharide macromolecules consisting of (α-1,4)-linked α-D-glucopyranose units, which are produced from the starch degradation by glucosyl transferase (6). As an alternative to native CDs, the chemically modified hydroxyalkyl derivative-hydroxypropyl-β-cyclodextrin (HP-β-CD) can specifically interact with food-derived compounds through the formation of non-covalently bonded units. Due to the truncated cone structure, the orientation of free hydroxyl groups toward the exterior of the cone imparts a hydrophilic peripheral contour, while the skeleton carbon and O atoms in the glycosidic bonds line the hydrophobic inner side cavity with lipophilicity. Therefore, HP-β-CD can be used to mask or reduce unpleasant smells and volatile chemicals, which have been widely applied in foods, agriculture, and the pharmaceutical field. Such non-covalent associations can increase the water solubility, bioavailability, and stability of the guest and can regulate the release of the guest molecules (7).

The beer foam stability is an urgent issue that needs to be solved during the brewing process. Quinoa saponins is a by-product of quinoa processing, which can also produce foam in an aqueous solution, and this foam is stable even at very low concentration (0.1%, taste tolerable). Therefore, quinoa saponins have potential application as a foaming agent (8–14). Takeshi et al. developed alcohol-free beer with saponins, indicating that the addition of saponins made beer drinks foam richer and more delicate (15). In the present study, a PC/HP-β-CD IC was prepared using a freeze-drying method and characterized by OM, TEM, UV, FT-IR, and TG/DSC methods. According to the principle of lowest energy, the possible conformations of IC were researched using HyperChem software 8.0. In addition, stability studies were conducted at different pH, temperatures, light conditions, and ethanol concentrations, and the total reduction ability and hydroxyl radical scavenging ability of the IC were evaluated. On this basis, quinoa saponins as foaming agents and IC as a colorant were explored to prepare blue beer, and the sensory properties, stability, and antioxidant properties of blue beer were evaluated. Therefore, this study supplied new thought for the development of blue beer products and the extension of their shelf life.

## 2. Materials and methods

### 2.1. Materials and reagents

Phycocyanin was obtained from TCI (Shanghai). HP-β-CD (>99%, average Mw = 1,460) was purchased from Sigma-Aldrich Shanghai Trading Co., Ltd. (China). The raw material used to brew beer, including Australian malt, domestic Magnum bitter hops (α-acids: 12%), Tsingtao Flower hops (α-acids: 6.6%), and Angel active dry yeast CN36, were purchased from Jinan Shuangmai Beer Materials Co., Ltd. (Jinan, China). All other reagents were of analytical grade.

### 2.2. Preparation of PC/HP-β-CD IC

PC/HP-β-CD ICs were prepared by the freeze-drying method according to previous research (16, 17). Briefly, 30 mg of PC and 600 mg of HP-β-CD were dispersed in 152 mL of an aqueous solution. The mixture was ultrasonically treated and magnetically stirred for 24 h at 25°C without light. After filtering through 0.45-μm filters, the reactant was lyophilized at −54°C for 8 h at 30 Pa using a freeze dryer. Finally, 524.5 mg of the resulting freeze-dried powder was obtained. Dark and cool storage conditions are needed.

### 2.3. Loading content and purity ratio

To calculate the loading content and purity ratio of the complex, we prepared ICs at different molar ratios and PC aqueous solutions with the method above (18). Absorbance spectra of gradient diluted ICs and PC with the final concentration of 0, 0.2, 0.4, 0.6, 0.8, and 1.0 mg/mL were measured using a UNICO 2100 UV-VIS spectroscope (Unico, Shanghai, China) at the maximum absorption wavelength from 200 to 800 nm. The regression equation was calculated based on the absorbance (*Y*) and the PC concentration (*X*). The loading content of the IC was given as the ratio of the amount of entrapped PC to the amount of the IC. The purity ratio of PC was defined as the ratio of *A*_620_*/A*_280_. The calculation equations were shown below.


(1) $$\begin{array}{l} Loading\;content\;\left(\%\right)\;=\;\frac{Amount\;of\;PC\;entrapped}{Amount\;of\;IC}\;\times \;100\%\; \end{array}$$

(2) $$\begin{array}{l} Purity\;=\;\frac{A_{620}}{A_{280}}\; \end{array}$$

where *A*_620_ is the absorbance of PC at 620 nm and *A*_280_ is the absorbance of total proteins.

### 2.4. Spectroscopy analysis

#### 2.4.1. Ultraviolet–visible (UV–vis) spectroscopy

At room temperature, PC (5 mg), HP-β-CD (10 mg), physical mixture (PC 5 mg and HP-β-CD 10 mg), and ICs (10 mg) are dissolved in 5 mL of distilled water, respectively. Then, the UV-vis spectra of PC, HP-β-CD, physical mixture, and ICs were monitored using a UV-vis spectrophotometer from 200 to 800 nm.

#### 2.4.2. Fourier-transform infrared (FT-IR) spectroscopy

FT-IR spectra of PC, HP-β-CD, physical mixture, and ICs were recorded using a Bruker Tensor II FT-IR Spectrometer (Karlsruhe, Germany) at the range of 400–4,000 cm^−1^. First, 100 mg of dried KBr and 1 mg of the tested sample were weighed, and the mixture was ground into fine powder in the sampling cup. Then, the power bed was compressed and loaded into a mold. Finally, the tested sample was mounted specifically in the light path and the corresponding spectrum was obtained.

### 2.5. Morphological and conformational characterization

OM and TEM were implemented to observe the microstructures and particle sizes of PC, ICs, and HP-β-CD. In mesoscopic characterization, the pretreated samples were assessed at 40 × on a BT-1600 image particle analyzer (Better, Dandong, Liaoning, China). TEM images were obtained using an HT7700 TEM meter (Hitachi, Tokyo, Japan) operated at the accelerating voltage of 80 kV. Before the test, the samples dissolved in alcohol were placed on a 0.085-mm carbon-coated Cu grid and dried. To ensure the reproducibility and representativeness of the tested samples, different images were observed and recorded from various parts of the samples.

### 2.6. Thermogravimetry (TG)/differential scanning calorimetry (DSC)

A TA SD TQ600 TGA/DTA thermal analyzer (New Castle, USA) was used to investigate the thermal properties of free PC, HP-β-CD, physical mixture, and ICs. Approximately 10 mg of a sample was placed on a tared aluminum weighing vessel and moved to the TGA furnace at room temperature. The testing temperature was raised from ambient temperature to 500°C at a heating rate of 10°C/min. All DSC measurements were conducted in a nitrogen atmosphere at a flow rate of 100 mL/min.

### 2.7. Assessment of IC stability

For the stability assessment, the pigment retention of PC was calculated spectrophotometrically. The pigment retention was determined according to Equation (3).


(3) $$\begin{array}{l} Pigment\;retention\;\left(\%\right)\;=\;\frac{A}{A_{0}}\;\times \;100\%\; \end{array}$$

where *A* and *A*_0_ are the final and initial absorption of PC, respectively.

The pH stability of IC was measured according to the previous method (19) with some modifications. Briefly, the PC and IC solutions were prepared in phosphate buffer at pH 2.0, 4.0, 6.0, 8.0, and 10.0. The samples diluted in deionized water (pH 7) served as the control.

The thermal stability of IC was measured according to the previous method (20) with some modifications. Briefly, the free PC and the IC solution were incubated at 4, 25, or 40°C for 24 h. To further investigate the effects of short-term heating incubation, we incubated the PC solution at 70 or 100°C for 30 min. The thermal stability was monitored and determined.

The ethanol stability of free PC or the IC was detected in 5%, 10%, 20%, 30%, 40%, and 50%, v/v ethanol concentrations at 25°C in the absence of light at room temperature. After 24 h of treatment, the retention amount of PC was measured. The sample diluted in deionized water (without ethanol) served as the control.

The light stability of IC was measured according to the previous method (21) with some modifications. The pretreated samples were placed in a 150-L constant-temperature incubator at 25°C and exposed to an 8-W fluorescent lamp (<500 nm) and an 8-W ultraviolet lamp (366 nm) at a distance of 20 cm for 24 h.

### 2.8. Antioxidant calculation

The antioxidant properties of PC and ICs were evaluated using a reported method (22–24) with minor modifications.

#### 2.8.1. measurement of reducing power

Briefly, 2.5 mL of a phosphate buffer (0.2 mol/L, pH 6.6) and 1% aqueous potassium hexacyanoferrate [K_3_Fe(CN_6_)] solution were mixed with 1 mL of tested extract. The mixtures were incubated at 50°C for 30 min, and then, 2.5 mL of 10% trichloroacetic acid was added to the mixture. After centrifuging, the upper layer was mixed with 2.5 mL of distilled water and 0.5 mL of 0.1% aqueous FeCl_3_, and the absorbance of the resultant solution was measured at 700 nm.

#### 2.8.2. Hydroxyl radical scavenging activity

Briefly, 1 mL of 1,10-phenanthroline (1.865 mmol/L) was mixed with 2.0 mL of sample solution. Then, 1.0 mL of FeSO_4_·7 H_2_O (1.865 mmol/L) and 1.0 mL of H_2_O_2_ (0.03%, v/v) were added sequentially. The reaction system was incubated at 37°C for 60 min. Afterward, the absorbance of the reaction solution was detected at 536 nm. The hydroxyl radical scavenging ability was measured as follows (4).


(4) $$\begin{array}{l} K\;\left(\%\right)\;=\;\frac{A_{s}-A_{n}}{A_{b}-A_{n}}\;\times \;100\%\; \end{array}$$

where *A*_*s*_ was the absorbance of the reaction solution contained samples, *A*_*n*_ was the absorbance of the reaction solution with the solution replaced by deionized water, *A*_*b*_ was the absorbance of the reaction solution with the sample solution, and H_2_O_2_ was replaced by deionized water.

### 2.9. Molecular modeling

The molecular modeling of the ICs was performed using a previous method with minor modifications (25). HyperChem 8.0 with MM2 force field method was calculated for the docking process. The original 3D structures of PC and HP-β-CD were built on HyperChem 8.0 and optimized with PM3. The optimized structure of phycobilin, the main component of PC, gradually entered the cavity of HP-β-CD from the wider edge and narrow edge separately. Both poses were minimized using a conjugate gradient optimization procedure until a root mean square (RMS) reached 0.01 kcal/(mol·Å). Δ*E* was calculated according to equation (5).


(5) $$\begin{array}{l} \Delta E_{c}=E_{complex}-\left(E_{host}+E_{guest}\right) \end{array}$$

where *E*_*host*_, *E*_*guest*_, and *E*_*complex*_ (kcal/mol) are the calculated energy of HP-β-CD, phycobilin, and the IC respectively.

### 2.10. Applications of HP-β-CD IC in the blue beer containing quinoa saponins

The extraction process of quinoa saponins was performed using a reported method with minor modifications (11). In detail, quinoa seed peel (20 g) was added to 100 mL of petroleum ether for degreasing, swirled for 2 min, and centrifuged at 2,900 rpm for 5 min. After centrifugation, 100 mL of ethyl acetate was added to remove flavanone, which was basically the same as the degreasing operation. The ultrasonic microwave-assisted extractor was used for extraction with 80% ethanol for two times. The parameters for extraction were as follows: microwave power was 80 W, ultrasonic frequency was 80 kHz, solid–liquid ratio was 1 g: 15 mL, temperature was 60°C, and extraction time was 60 min. The ultrasonic extraction solution was combined and filtered through a 0.45-μm filter membrane. After filtering, the total saponin quinoa powder was obtained after rotary evaporation and freeze-drying methods.

Beer was prepared according to the previous method (5) and labeled as sample I. Afterward, quinoa saponins (4.5 mg/300 mL) were added to sample I to obtain sample II. Phycocyanin (20.7 mg/30 mL) was added to sample II to obtain sample III. An inclusion complex (445.0 mg/30 mL) was added to sample III to obtain sample IV. Each sample was evaluated for algal taste, bitterness, and antioxidant activity. The effect of light and temperature on the color was assessed in sample III and sample IV.

### 2.11. Statistical analysis

All of the data were measured in triplicate and presented as mean ± standard deviation (SD). Statistically significant differences were used for multiple comparisons on SPSS statistic 22.0. A difference was significant at a *p* < 0.05.

## 3. Results and discussion

### 3.1. Loading content and purity ratio

Loading efficiency is the amount of the guest loaded per unit weight to the host and indicates the mass percentage of the host that is due to the encapsulated guest. The standard curve for the PC content was plotted spectrophotometrically. The regression equation of PC content (*X*) and the absorbance (*Y*) at 617 nm was *Y* = 1.4863X – 0.0118 with a good relationship (*R*^2^ = 0.9991). The calculated loading content of the IC was 4.65 ± 0.14%, which indicated the effective entrapments of PC by HP-β-CD. The freeze-drying method has powerful entrapment efficiency in comparison with other methods. In addition, the pigment purity of the IC was 1.67 ± 0.01. Though the purity decreased compared with the equal concentration of PC (2.71 ± 0.02), it can meet the demand of the food industry.

### 3.2. UV–vis spectra

**Figure 1:**
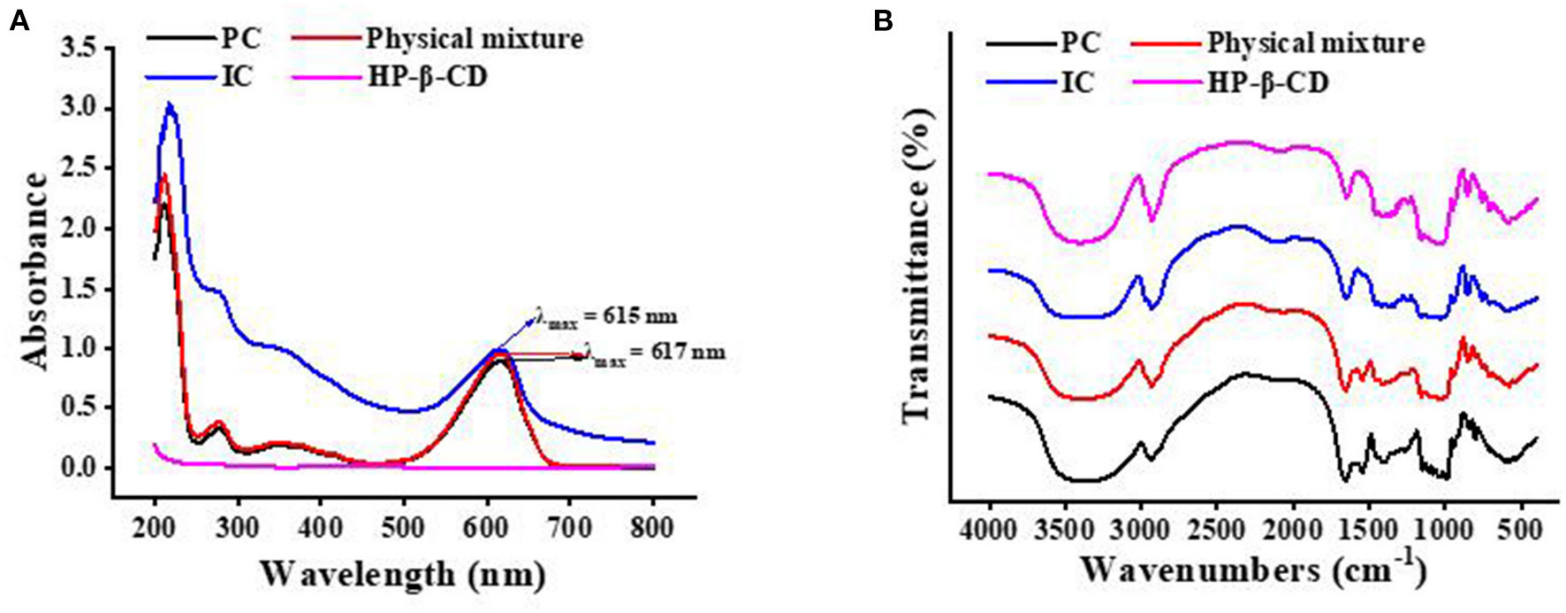
The ultraviolet–visible spectroscopy **(A)** and Fourier transform infrared spectroscopy **(B)** of the phycocyanin (PC), the physical mixture, the inclusion complex (IC), and hydroxypropyl-β-cyclodextrin (HP-β-CD).

As shown in Figure 1A, the UV/Vis spectra of PC, HP-β-CD, physical mixture, and ICs were recorded separately. HP-β-CD had no absorption in the range of 200–800 nm, which is in agreement with a previous report (26). Phycocyanin had three absorption peaks in the scanned range, in which the highest peak at 617 nm represents the band absorption generated by the conjugated system of the chromophore in PC. The characteristic peak at 278 nm was generated by unsaturated bonds of tryptophan and tyrosine residues. The weak peak at 346 nm was generated by the tetrahydropyrrole group of the whole molecule of PC, which may be related to the disulfide bonds in the PC. In addition, though the absorption peaks at 278, 346, and 617 nm increased slightly in intensity, the physical mixture of PC and HP-β-CD has a similar spectrum as PC. The spectra of the IC obviously show that the three peaks at 278, 346, and 617 nm blue-shifted and increased significantly. The reasonable explanation was that HP-β-CD formed a complex with PC, as reflected in the change of absorption peaks.

### 3.3. FT-IR spectra

The changes in the IR peaks of the guest, host, and IC can supply abundant information about the formation of the inclusion (27). Figure 1B shows the FT-IR spectra of PC, HP-β-CD, physical mixture, and the IC. The IR spectrum of pure PC in Figure 1B shows the characteristic strong bands at 3,357 cm^−1^ for N-H and O-H stretching vibration, at 2,929 cm^−1^ for methyl and methylene stretching vibration, at 1,656 cm^−1^ for C=O stretching vibration, at 1,546 cm^−1^ for C=C and C-N stretching vibration, at 1,397 cm^−1^ for carboxyl stretching vibration, at 1,257 cm^−1^ for C-N in-plane bending vibration and N-H stretching vibration, and at 611 cm^−1^ for C=O in-plane bending vibration. Apparently, the IR spectrum of the physical mixtures includes the characteristic peaks of PC and HP-β-CD. Specifically, the characteristic peak of PC disappeared at 1,546 cm^−1^, and the peak at 1,397 cm^−1^ intensity slightly decreased after the formation of the ICs. This can be also due to the restriction of C=C and C-N stretching vibration of PC inside the CD cavity and to the low content of the guest in the complex (28). These changes explain that intermolecular hydrogen bonds play a crucial role during IC reorganization and formation.

### 3.4. Morphological and conformational characterization

**Figure 2:**
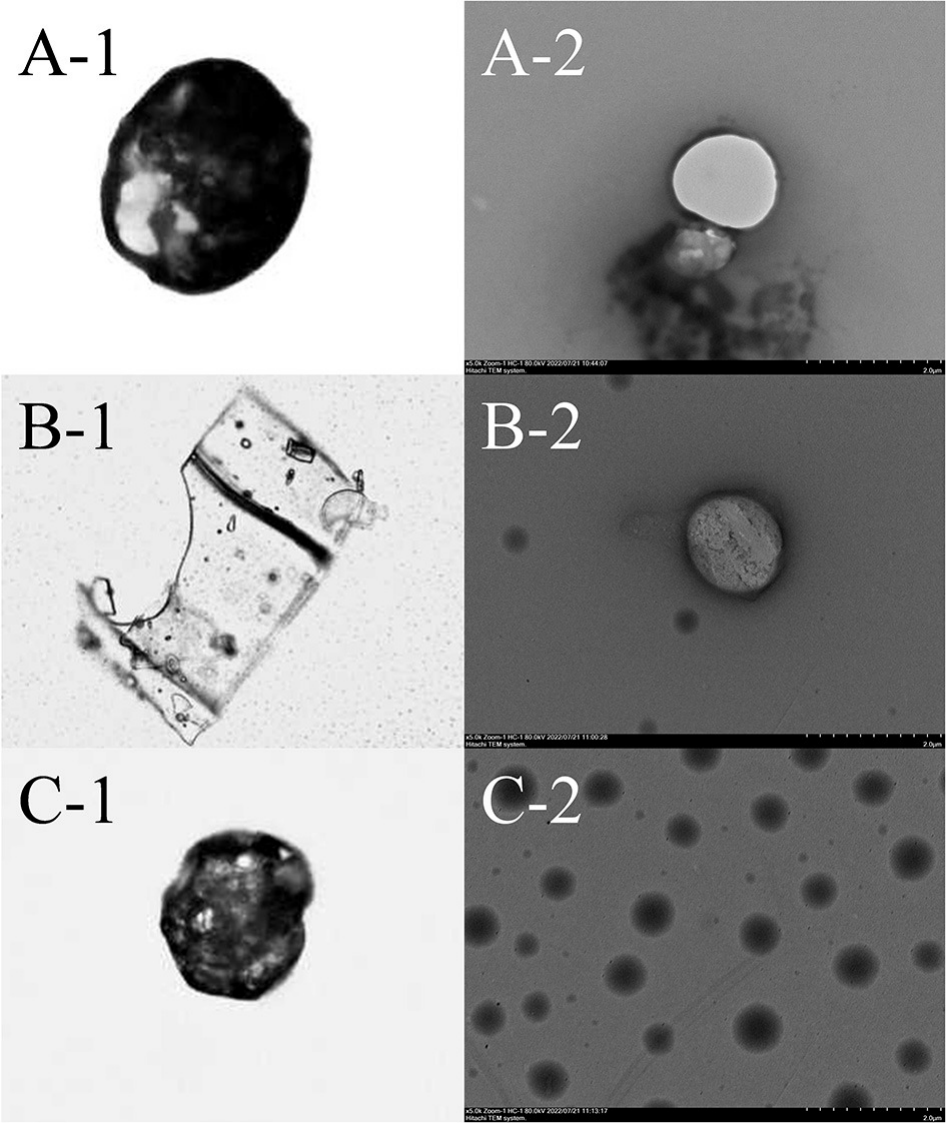
Optical microscopy (OM) and transmission electron microscope (TEM) images of PC **(A)**, IC **(B)**, and HP-β-CD **(C)**. Images on the left depict samples at 40 times magnification by OM. Images on the right depict samples at 5,000 times magnification by TEM.

Photomicrographs of PC, the ICs, and HP-β-CD detected using OM are presented in Figures 2A1–C1. HP-β-CD presents a rough surface with porous micropores and surface dimples. By comparison with free HP-β-CD, the ICs present irregular lamellar structures, indicating that HP-β-CD may contribute to the formation and agglomeration of complexes. This result is consistent with previous studies (29). TEM images of PC, ICs, and HP-β-CD are presented in Figures 2A2–C2. The free HP-β-CD shows smooth surfaces without cracks or pores. However, after the complex formation, there is a trend of agglomeration of ICs due to the existence of PC in the system, which is consistent with the previous study (30).

### 3.5. Thermal behavior analysis

**Figure 3:**
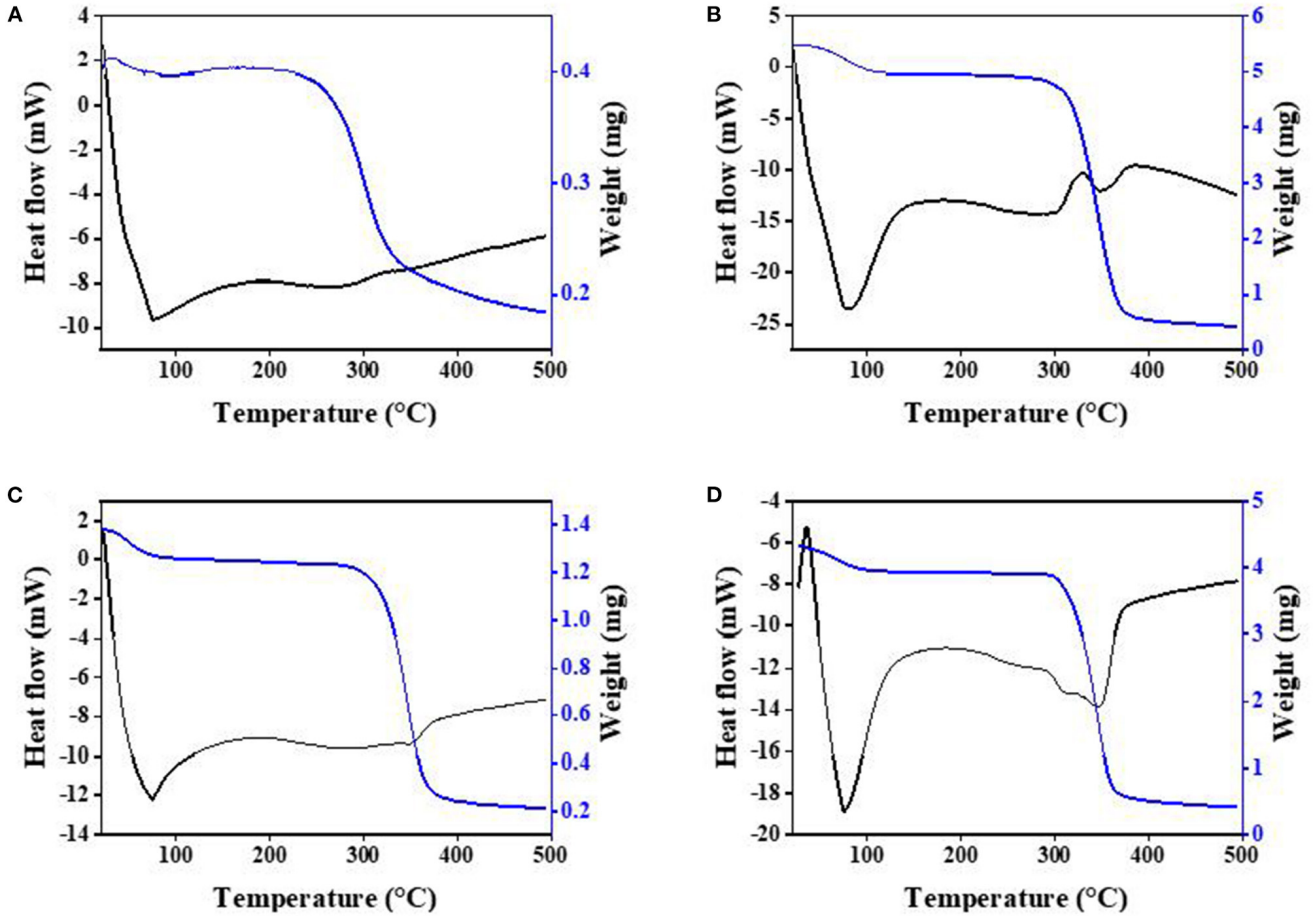
Thermogravimetry/differential scanning calorimetry (TG/DSC) curves of the PC **(A)**, the physical mixture **(B)**, the IC **(C)**, and HP-β-CD **(D)**.

DSC was used to recognize the ICs formed between HP-β-CD and the different guests. When the guest molecules were embedded into host cavities, their melting, boiling, or sublimating points generally shifted to different temperatures or disappeared (31). Figure 3 depicts the TG/DSC thermograms of PC, HP-β-CD, their physical mixtures, and ICs. The thermogram of pure PC in Figure 3A presents two endothermic bands at approximately 77 and 260°C. The first one is associated with the endothermic denaturation and water molecule evaporation of PC, resulting in the mass reduction of PC, as seen in the TG analysis. The main thermal decomposition stage of PC appeared within 200–340°C, with a peak occurring at 260°C. For HP-β-CD (Figure 3D), two broad endothermic peaks were observed approximately 312 and 348°C, which were associated with the thermal decomposition of HP-β-CD. The main mass loss phase within 300–370°C corresponds mainly to the high-temperature thermal degradation of HP-β-CD.

The DSC patterns of the physical mixtures (Figure 3B) present an endothermic peak at 350°C and a wide endothermic band at 260–310°C, which basically represent both pure PC and HP-β-CD peaks with slight shifts. These curves also indicate that no inclusion occurred when HP-β-CD and PC were simply mixed together. The TG curve of ICs in Figure 3C shows that the weight of the ICs continued to lose within the range of 280–380°C, and there was a less obvious endothermic peak near 347°C, which is different from the physical mixture of HP-β-CD and PC, further confirmed that the formation of ICs is not a simple physical mixing process. In addition, the thermal decomposition of the ICs was delayed by at least 80°C from the TG/DSC curves, indicating that PC was protected by the formation of the ICs with HP-β-CD.

### 3.6. Stability analysis of IC

**Figure 4:**
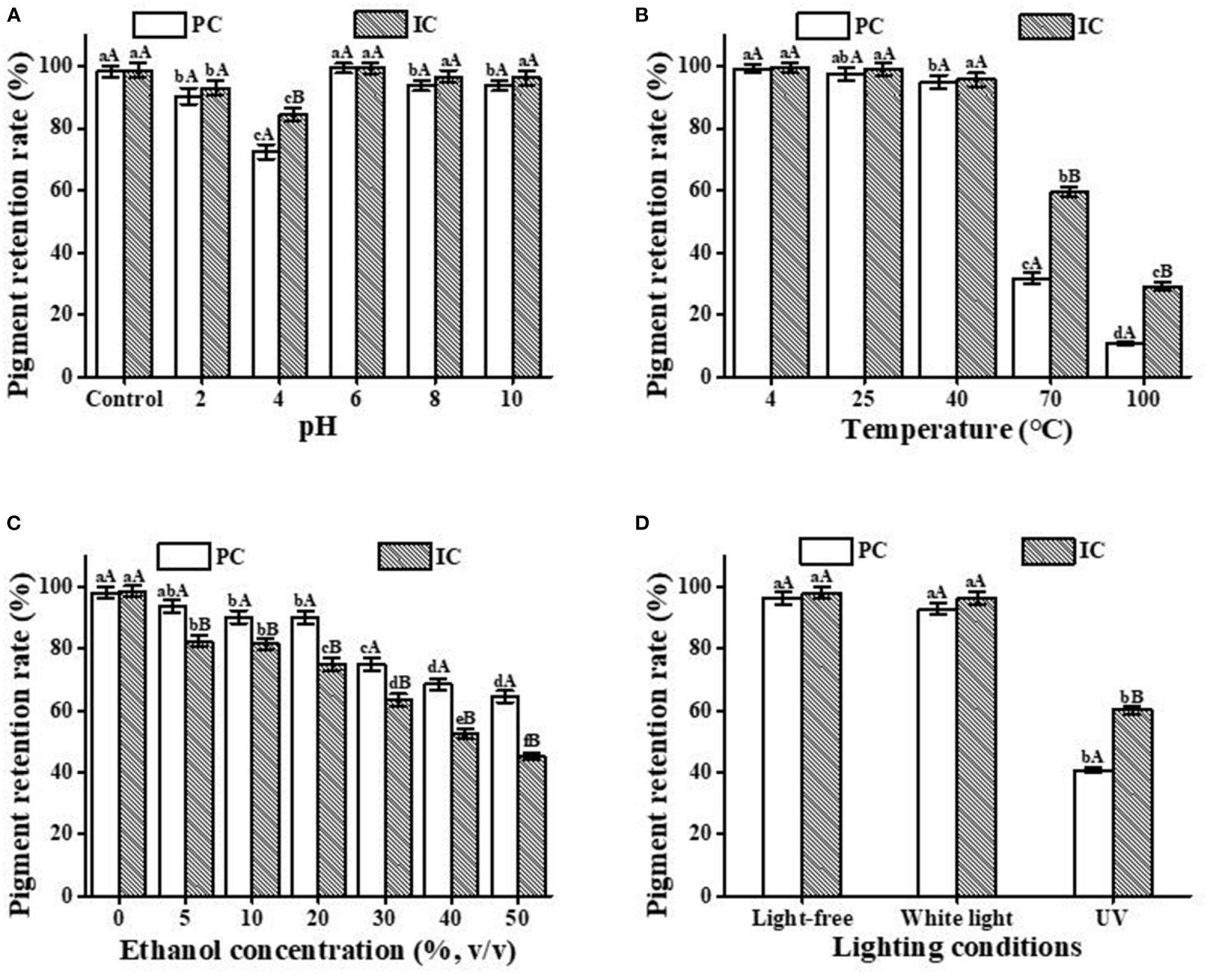
Stability analysis of the PC and the IC. **(A)** pH; **(B)** temperature; **(C)** ethanol concentration; **(D)** light condition. Lowercase letters represent significant differences between different treatment groups for the same sample, and capital letters represent significant differences between different samples within the same treatment group (*p* < 0.05).

The effect of HP-β-CD on the stability of PC is illustrated in Figure 4. High degradation occurred at pH 4, which may be close to the isoelectric point of PC. Compared with other pHs, the highest retention of PC occurred at pH 6. The degradation extent of ICs was slower compared with free PC, as clearly shown in Figure 4A. The results obviously indicate that the pH stability of PC is improved after inclusion complexation with HP-β-CD.

Figure 4B shows the thermal stability of free PC and IC in aqueous solutions at different temperatures. The pigment retention rate of free PC and IC were basically in higher level at 4, 25, and 40°C, which indicating that the storage conditions for both of PC and IC should be under 40°C. However, after heating at 70 and 100°C for only 30 min, the percentage of PC that remained in the solutions of both free and complex forms remarkably decreased compared with the low-temperature conditions. Therefore, in aqueous solutions at 70 and 100°C, HP-β-CD has a great protective effect on PC. This result has reference significance for PC to maintain pigment stability under a heating environment.

Figure 4C shows that when exposed to different concentrations of ethanol, free PC and the ICs were gradually degraded. In particular, when the ethanol concentration was higher than 30%, the retention rate was only 80% after treatment for 24 h. However, the degradation of PC in the complex form occurred at a quicker rate, which can be explained by the fact that the solubility of HP-β-CD in ethanol is significantly higher than that of PC. This will result in more PC exposure to ethanol, which may cause denaturation of PC. Therefore, the application of PC/HP-β-CD ICs into cocktails or other alcoholic beverages will be no more than 30% of ethanol.

Photodegradation of compounds can be hindered through host/guest IC formation. To evaluate the photostability of the free PC and the IC, we exposed the samples to UV and fluorescent lamps for 24 h. Figure 4D shows that due to the protective effect of HP-β-CD, the retention rates of PC and IC basically reached the same level as the light-free and the white light condition, suggesting that HP-β-CD presented a great protective effect on the white light exposure. However, compared with the light-free and white light conditions, the retention rate of PC decreased significantly after exposure to UV light for the same period. The formation of ICs has a particularly significant protective effect on PC from ultraviolet radiation. Therefore, HP-β-CD can significantly improve the photostability of PC and provide a reference for food storage and transportation.

### 3.7. *In vitro* antioxidant capacity

**Figure 5:**
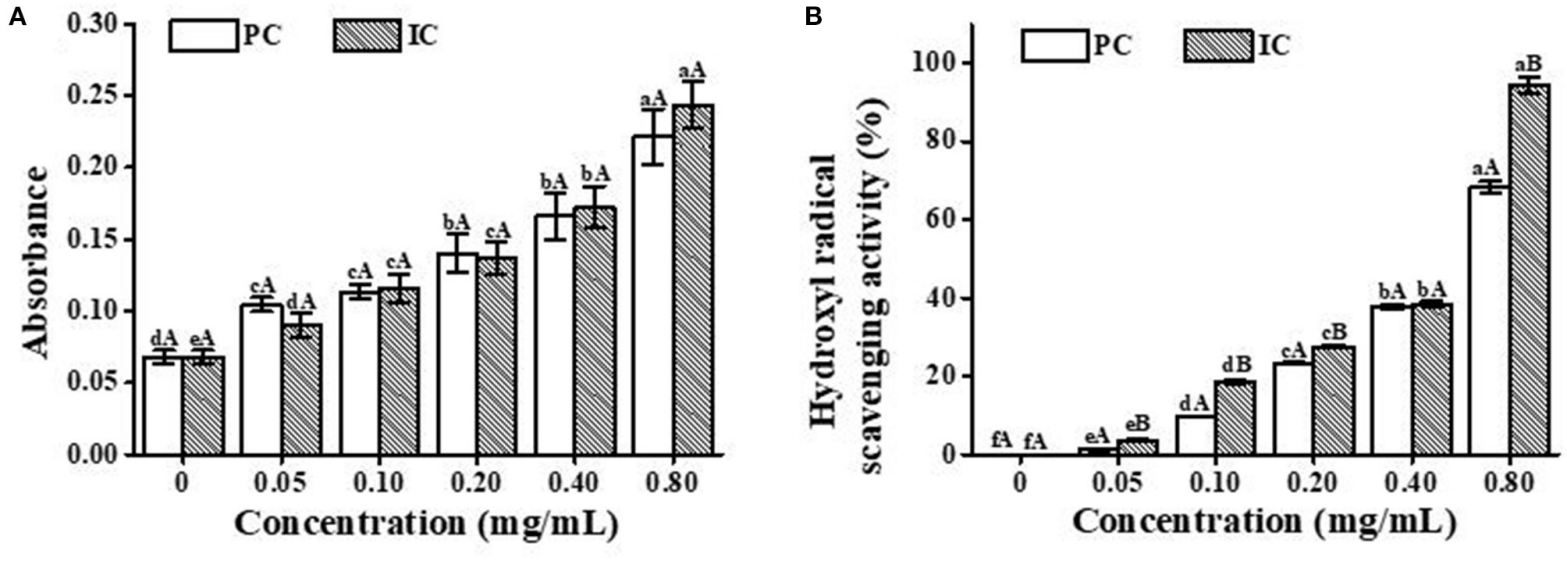
Antioxidant activity of PC and the IC *in vitro*. **(A)** is the total reduction capacity, and **(B)** is the hydroxyl radical scavenging activity. Lowercase letters represent significant differences between different concentrations for the same treatment group, and capital letters represent significant differences between different treatment group samples within the same concentration (*p* < 0.05).

For both PC and ICs, the notable dose-dependent reducing ability was generally observed in Figure 5A. In the low-concentration groups, free PC presented higher antioxidant activity than the ICs. A reasonable explanation was probably that the relative concentration of PC in the reaction systems decreased with the content increase in ICs, in which HP-β-CD hampered the release of PC. In the high-concentration groups, however, the ICs were observed with better antioxidant activity compared with the free PC, which may be ascribed to the protective effect of HP-β-CD (32). Figure 5B shows that the hydroxyl radical scavenging activities of both free PC and ICs were enhanced with the increase in concentration. With the high concentration of PC (0.8 mg/mL), the antioxidant activity of ICs was obviously stronger than the pure PC, which represents the protective effect of HP-β-CD.

### 3.8. Molecular modeling

**Figure 6:**
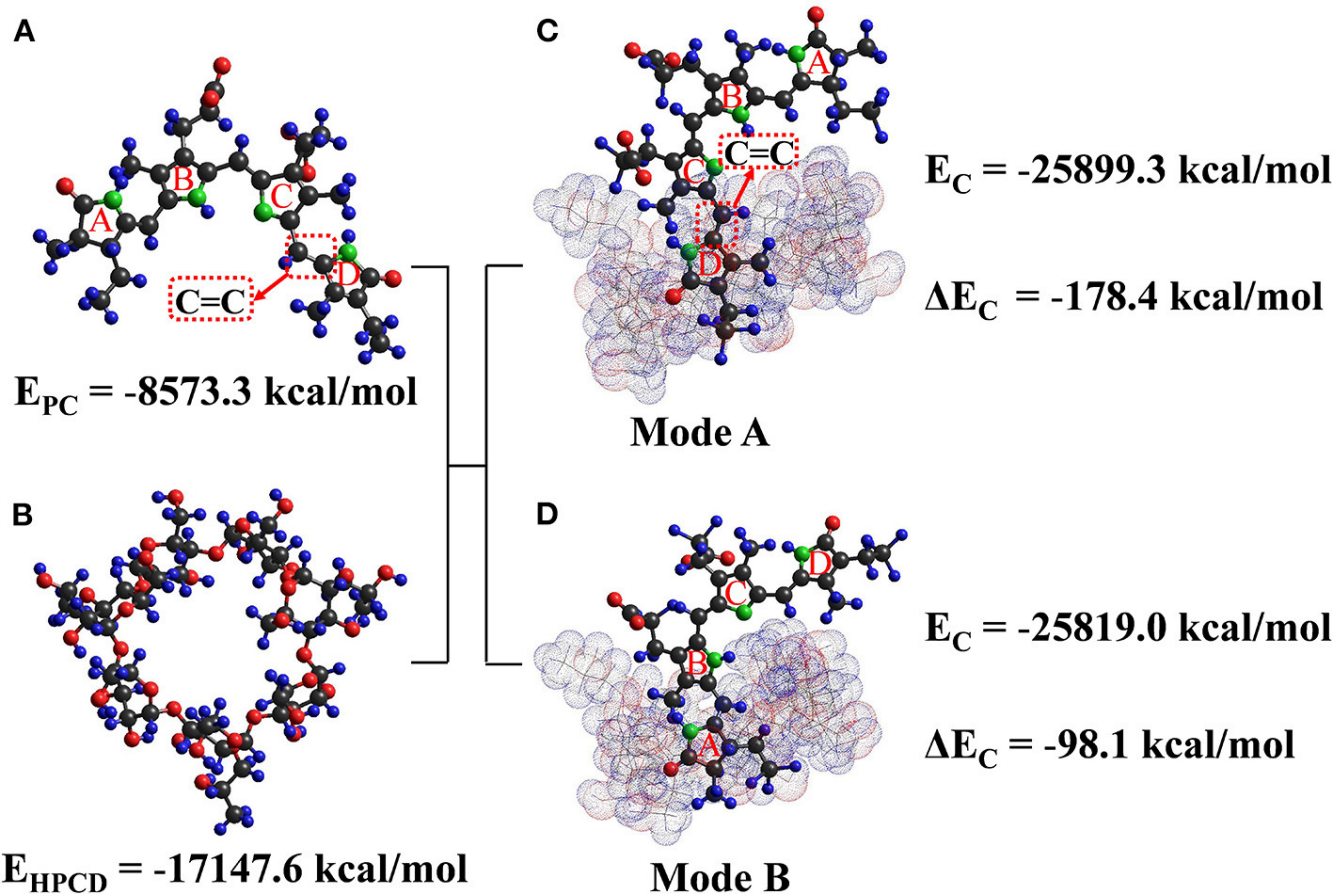
The energy-optimized molecular models of phycobilin, HP-β-CD, and the IC. **(A)** is the 3D structure of phycobilin, **(B)** is the 3D structure of HP-β-CD, **(C)** is binding mode a of IC, and **(D)** is binding mode b of IC.

Molecular docking is a strong tool for elaborating the complexation mechanism between HP-β-CD and PC. The optimal geometry and specific molecular interactions of ICs were calculated based on the PM3 method (33). The chromophore in the PC molecule is an open-chain tetrapyrrole structure (four pyrrole rings A, B, C, and D) (34). The guest was not completely embedded in the hydrophobic inner side cavity of the host in either mode (Figure 6), where Δ*E*_*c*_ of mode a and mode b was −178.4 and −98.1 kcal/mol, respectively, highlighting the stable and favorable interaction between the guest and host molecules. As the lowest total energy was shown by 1:1 stoichiometry of the guest: host IC of mode a, it was considered to be the most stable IC between the two modes. Specifically, the D-pyrrole ring of PC and its associated C=C group entered the HP-β-CD cavity from the wider edge, while ring C was close to the wide end. It can also be speculated that hydrogen bonding and van der Waals interactions play a stabilizing role in the ICs formation, which thus corroborates the conclusions drawn from the FT-IR spectra.

### 3.9. Improved stability of PC in the blue beer containing quinoa saponins

**Figure 7:**
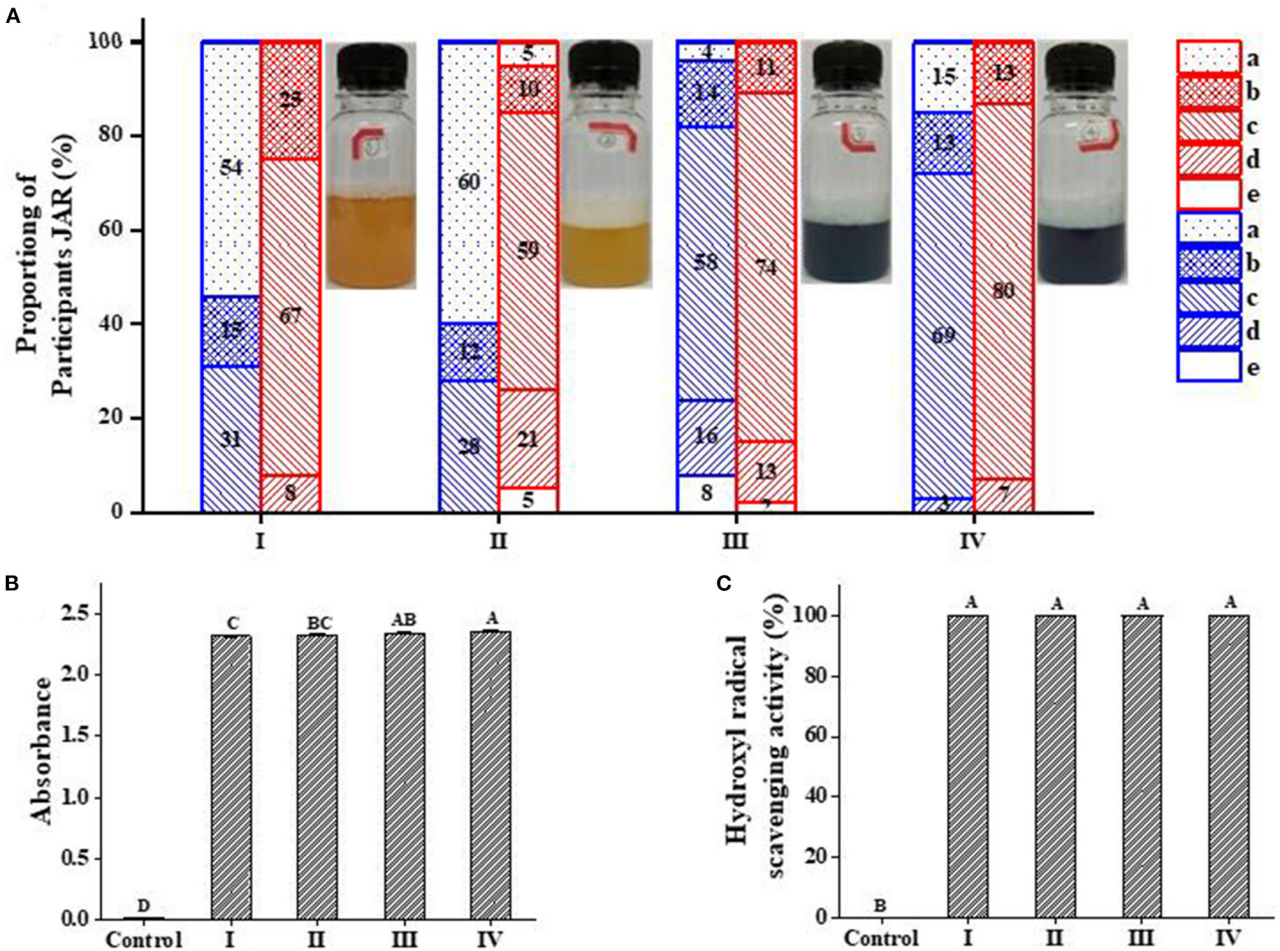
Just-about-right (JAR) frequencies for the attributes of algal taste and bitterness and antioxidant activity of beer samples (I, II, III, and IV). **(A)** Blue, algal taste; red, bitterness; a-e represent much too little, little too little, just right, little too much, and much too much, respectively. **(B)** is the total reduction capacity; **(C)** is the hydroxyl radical scavenging activity. **(B, C)** Capital letters represent significant differences between different samples within the same treatment group (*p* < 0.05).

The flavor evaluation results of the beer sample were shown in Figure 7A. Because PC has its own special flavor, the algal taste was less inconspicuous in sample III and sample IV while sample IV showed better overall acceptance. Besides this, the addition of quinoa saponins makes the foam of beer more delicate and it also increased the bitter taste of beer. The addition of PC weakened the bitterness of beer, while the IC showed a stronger weakening effect. The reason for the above results may be that the cyclodextrin in the ICs had a taste-masking effect on algal taste and bitterness (35, 36).

Figures 7B, C shows that the four beer samples showed strong reducing power capacity and hydroxyl radical scavenging ability. This result is obviously superior to the antioxidant capacity corresponding to the maximum concentration of the sample shown in Figure 5. This phenomenon is not only related to the antioxidant properties of PC, but also related to the polyphenols in barley malt and the antioxidant properties of quinoa saponins (37–40). This result can provide theoretical support for the blue beer promotion.

**Figure 8:**
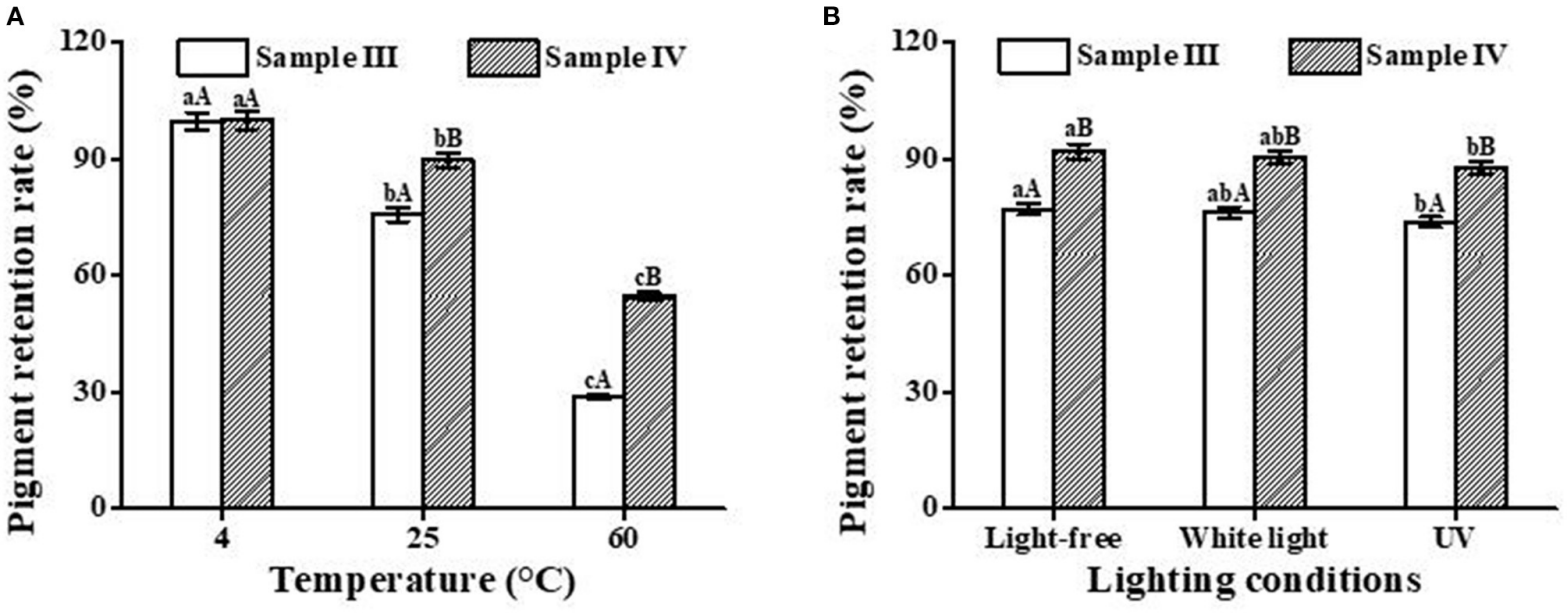
Effects of temperature **(A)** and light conditions **(B)** on the color stability of blue beer samples (III and IV). Lowercase letters represent significant differences between different treatment groups for the same sample, and capital letters represent significant differences between different samples within the same treatment group (*p* < 0.05).

Figure 8A shows that temperature has a significant effect on the retention rate of PC in blue beer. The retention rate decreased with the increase in temperature. At the same temperature, the retention rate of sample IV is higher than that of sample III. This is likely related to the protective effect of cyclodextrin in the IC. As shown in Figure 8B, light-free and white light have little impact on the stability of the pigment in beer samples, which is similar to the result from Figure 4D. However, different from the abovementioned result, ultraviolet radiation has no destructive effect on the stability of pigment in beer samples, which may be related to the polyphenols contained in barley malt (41, 42).

## 4. Conclusion

An IC of PC and HP-β-CD was developed using a freeze-drying method. The multi-spectroscopy analysis together with the characterization data from morphological and conformational observation showed that PC could merge into the cavity of HP-β-CD. Then, the potential of HP-β-CD to protect PC from heat, pH, ethanol, and light exposure was investigated. The different types of processing stability (except ethanol) of PC were greatly improved after the formation of ICs with HP-β-CD. Moreover, the *in vitro* test showed that the ICs presented a remarkable antioxidant capacity. Molecular modeling results clearly demonstrated that PC can be efficiently complexed with HP-β-CD to form an IC at a molar ratio of 1:1. The D-pyrrole ring of PC and its associated C=C group entered the HP-β-CD cavity from the wider edge. In conclusion, ICs can be a promising strategy in food and beverage coloring, which thereby expands the potential application of PC. Furthermore, the results of this study show that it is feasible to use quinoa saponins as the foaming agent of beer, and the encapsulation effect of cyclodextrin can effectively improve the temperature stability and light stability of blue beer. This study can provide a basis for the development and promotion of blue beer.

## Data availability statement

The raw data supporting the conclusions of this article will be made available by the authors, without undue reservation.

## Author contributions

GZ: investigation and writing—original draft. HZ: investigation and software. TG: writing—review and editing. ZM: conceptualization and writing—review and editing. All authors contributed to the article and approved the submitted version.
